# Community-based health financing: empirical evaluation of the socio-demographic factors determining its uptake in Awka, Anambra state, Nigeria

**DOI:** 10.1186/s12939-021-01574-4

**Published:** 2021-10-26

**Authors:** Felix O. Iyalomhe, Paul O. Adekola, Giuseppe T. Cirella

**Affiliations:** 1 Department of Biological and Environmental Science, Kampala International University, 20000 Kampala, Uganda; 2 Department of Environmental Sciences, National Open University of Nigeria, Abuja, 900211 Nigeria; 3 Polo Centre of Sustainability, 18100 Imperial, Imperia Italy; 4 Demography and Social Statistics Programme, Department of Economics and Development Studies, College of Management and Social Sciences, Covenant University, Ota, 112233 Nigeria; 5 Centre for Economic Policy and Development Research, Covenant University, Ota, 112233 Nigeria; 6 Faculty of Economics, University of Gdansk, 81-824 Sopot, Poland

**Keywords:** Healthcare, Health schemes, Community fund, Prevention, Sub-Saharan Africa

## Abstract

**Background:**

There is an increasing global concern of financing poor people who live in low- and middle-income countries. The burden of non-communicable diseases of these people is, by in large, connected to a lack of access to effective and affordable medical care, weak financing, and delivery of health services. Policymakers have assumed, until recently, that poor people in developing countries would not pay health insurance premiums for the cost of future hospitalization. The emergence of community-based health financing (CBHF) has brought forth a renewed and empowered alternative. CBHF schemes are designed to be sustainable, varying in size, and well organized. Developing countries, such as Nigeria, have been testing and finetuning such schemes in the hope that they may 1 day reciprocate high-income countries.

**Methods:**

A sample size of 372 respondents was used to assess the slums of Awka, the capital city of Anambra State, Nigeria, and empirically evaluate the socio-demographic characteristics of those who uptake CBHF using the provider Jamii Bora Trust (JBT). Cross-sectional research used a quantitative research approach with the instrumentality of structured questionnaires. Descriptive analysis was adopted to determine the socio-demographic characteristics of those who have CBHF uptake in Awka and evaluate the presence and benefits of CBHF in the city’s slums.

**Results:**

The results show that more youth and middle-aged persons from 18 to 50 years are more insured (i.e., 73.8% combined) than those who are over 50 years of age. Gender distribution confirm more females (i.e., 61.9%) to be health-insured than their male counterpart (i.e., 38.1%). This perhaps reflected the reproductive roles by women and the fact that women have better health-seeking behavioral attitude. Moreover, the results correlate with previous studies that confirm women are more involved in local sustainable associations in low-income settings, of this nature, in sub-Saharan Africa. Corroborating this further, married people are more insured (i.e., 73.8%) than those who are not married (i.e., 26.2%) and insured members report higher use of hospitalization care than the non-insured.

**Conclusion:**

CBHF uptake favored members in the lower income quintiles who are more likely to use healthcare services covered by the JBT scheme. This confirmed that prepayment schemes and the pooling of risk could reduce financial barriers to healthcare among the urban poor. Recommendations are suggested to improve enrollment levels in the CBHF programs.

## Introduction

One of the increasing concerns of promoting global universal health has been financing for more than 1.5 billion poor people who live in middle- and low-income countries [1, 2]. The burden of non-communicable diseases is on the rise in developing countries and a large percentage of the poor lack access to effective and affordable medical care due to weakness in the financing and delivery of health services. One of the major challenges facing many developing countries is how to provide healthcare for the poor who live in rural areas or work in the informal sector. The burden of disease in these countries stands as a stark barrier to economic growth and therefore must be addressed frontally and centrally in any comprehensive development strategy. This has prompted the demand for community-based health promotion models that focus on transaction and internal costs [3]. According to the World Health Organization (WHO), 150 million people in 44 million households globally face financial ruin as a direct result of large medical bills annually [2]. Policymakers have assumed, until recently, that poor families in developing countries whose survival is vicarious would not pay health insurance premiums, even to forestall the cost of future hospitalization [2, 4, 5]. However, the emergence and thriving concept of community-based health financing (CBHF) has proven that the poor, if empowered, can participate in sustainable health schemes [6–10]. Well organized and sustained small CBHF schemes can develop to become strong and acceptable social health insurance systems. This is true of the health insurance systems currently operating in Germany [11], Japan [12], and South Korea [13, 14]. Today’s CBHF schemes are operated in a manner similar to the societies which existed, in large numbers, in the United Kingdom during the nineteenth century [15]. CBHF aims to empower communities to meet their health financing needs through pooling of resources to pay for healthcare as a group. The schemes share the goal of finding ways for communities to meet their health financing needs through pooled revenue collection and resource allocation decisions made by the community [16]. Schemes employ a variety of financial structures including insurance, prepayment, and credit schemes with premiums ranging from a once per annum payment during harvest season to a monthly or quarterly fee [10]. CBHF schemes can act as a resource to pay for services through a community fund or be facility-based [17].

Community-based health promotion models target the whole community instead of disease-based models in which only the patient is targeted for treatment [18–20]. Although many community-based health promotion interventions have been implemented in different countries, a holistic commitment for global health promotion was launched at the First International Conference on Health Promotion held in Ottawa, Canada, in 1986 [21]. This conference resulted in the Ottawa Charter which defined health promotion as the “process of enabling people to increase control over and to improve their health” [21]. The Charter also advocated creating healthy environments in schools, hospitals, workplaces, and community dwellings. It emphasized that there is a health development potential in every organization and every community; it submitted the main focus of community empowerment is health promotion activities. It has been argued that illness reduces not only welfare but also increases the risk of impoverishment due to high treatment expenditure in the absence of health financing [15, 17, 20]. Subsequently, households often resort to leaving the illness untreated or resort to self-medication [22–25]. There is a need for the health financing system to be developed within the particular macroeconomic, socio-cultural, and political context of each country. It should create balanced incentives with regard to equity, efficiency, sustainability, and quality of care. The collaboration between governments and development partners should follow internationally respected principles of the Paris Declaration of 2005 [26] and, thus, ensure national ownership of the health development policies and processes to maximize limited resources and reduce transaction and management costs [27].

CBHF schemes can be used to assist the health status of many developing countries and have been found to be cost effective and replicable. In the case of Nigeria, the principal healthcare financing agents are the households which belong to CBHF schemes through out-of-pocket payments. Given the social, physical, and demographic infrastructure in the country, such schemes become an integral part of informal settlements. The socio-economic status—characterized by poverty, poor housing, and inaccessibility to basic healthcare—underpin the essence of assessing the performance of any scheme. The purpose of the study is to undertake an empirical evaluation of the socio-demographic characteristics of those who participate in CBHF schemes uptake in Awka, Anambra State, Nigeria. This will help to provide national and state government with knowledgeable insight into the core features of developing robust policy guidelines in terms of management and scheme development. The finding will also enrich existing knowledge and, hence, will be of interest to academicians, health practitioners, insurance companies, and the broader community who seek to explore and carry out further investigations in this field of study.

## Literature review

### Origin of CBHF

Although the CBHF schemes have been considered as an innovative financing mechanism for the poor, they are not a new concept. Today’s social health insurance systems in many developed countries (e.g., Belgium and Canada) as well as tax-based financing schemes (e.g., Australia and Italy) date back to small-scale community-based schemes that would meet the definition of CBHF [10]. In some Asian countries, such as Thailand [28, 29] and China [30, 31], CBHF initiatives came about independently throughout the 1970s and 1980s to help fill in the gaps in coverage of public financing mechanisms. These schemes provided improved access to services and income protection measures for people in the informal sector in both rural and urban areas. Germany, Japan, Belgium, and the United Kingdom have reached or almost reached universal coverage in terms of depth (i.e., benefit coverage) and breadth (i.e., population coverage). Rather than being locally initiated by the informal sector as in Asia, or by the associations of industrial workers or employers in Europe, CBHF schemes in Africa are mostly the imported interventions of foreign aid agencies or national governments [32]. These schemes try to mitigate the effects of increases in user fees, the collapse of healthcare services in certain areas, and the difficulty of expanding formal health insurance to the informal sector. Both foreign aid agencies and national governments hope that CBHF will serve as a mechanism for the transition to universal coverage; however, no one society is the same as research across Africa indicates an array of variance.

For example, the Abota village insurance scheme in Guinea-Bissau was initiated in 1980 in the face of the breakdown of government funding for healthcare [33]. In contrast, all CBHF schemes that currently exist in Benin, Mali, Ghana, and Nigeria were founded in the 1990s [16, 32, 34–36]. In Ghana and Nigeria, they originated from mission hospitals in search of new sources of finance during a time of reduced government subsidies and declining external support, i.e., after the practice of levying user fees had proven to be inadequate [35]. Some of the schemes are confined to a local cooperative group (e.g., craftsmen or traders) and often are very small and may cover less than 100 beneficiaries [37]. Other CBHF extend over many communities and can include up to a million, or even more, beneficiaries [16]. The number of beneficiaries can change rapidly and neither reveals the financial balance or sustainability of the scheme. As such, a number of schemes have been terminated [16, 32] whilst others have been in operation for decades [38].

In Nigeria and Kenya, several CBHF schemes have emerged to meet the healthcare financing needs of low-income earners who have traditionally been largely left out of the private insurance system. CBHF schemes vary greatly in type and scope and range from small funds run by community welfare groups to large non-governmental organization-based schemes. In Kenya, CBHF schemes have formed an umbrella association known as the Kenyan Community Based Health Financing Association (KCBHFA) in which organizations are registered under the Ministry of Gender and Youth. In Nigeria, nine primary organizations similarly based on the KCBHFA system intercorrelate throughout the country, namely: Western Region Community Services, Christian Organizations Research and Advisory Trust for Africa, Tropical Institute of Community Health, Christian Health Association of Nigeria, International Center for Development and Research, Eldoret Christian Community Services, Inter-Diocesan Christian Community Services, Support for Tropical Initiatives in Poverty Alleviation, and Jamii Bora Trust (JBT). Remarkably, JBT, a microfinance institute, was founded by Ingrid Munrowith’s initial small loans to fifty beggars in Nairobi in 1999 and, to date, has grown into one of the largest microfinance institutions in Africa [39]. JBT has over 280,000 members and 90 offices. Its largest member base is in the slums of Kibera, outside Nairobi, Kenya, and Awka, Anambra State, Nigeria. Small loans, even as small as a few American dollars, are given as start capital for small businesses and for medical clinics.

### Empirical evidence of CBHF in Africa

Many sub-Saharan countries began the post-colonial period with the intention of providing free or heavily subsidized healthcare to their citizens. Difficult economic periods, during the 1970s and 1980s, resulted in the abandonment of a universal health goal by virtually all sub-Saharan African countries. In the late 1980s, African health ministries, guided in part by the United Nations International Children’s Emergency Fund (UNICEF), the World Bank, and WHO, shifted their healthcare strategies to a subsidized, fee-for-service model that involved active community participation [36]. Launched in Bamako, UNICEF and a group of African health ministers initiated the Bamako Initiative in 1987, calling for greater community involvement at the local level. In the 1980s, the imposition of user fees at public health facilities across many health systems in sub-Saharan Africa resulted in diminished levels of access to healthcare services. In some ways, CBHF schemes can be viewed as a means of implementing one of the central tenets of the Bamako Initiative, i.e., local community member involvement both in the collection and control of healthcare revenue [36]. During this same period, some groups began to experiment with local health financing schemes in large part because of the breakdown in government health services and need to find alternative ways to finance local health services. These schemes can be divided into three main categories: formal sector (i.e., often employment-related) schemes, clan or ethnic-based schemes, and provider community schemes. In spite of this, schemes controlled by groups of rural community residents did not figure prominently in the programs until the late-1990s [40].

Mali was the first West African country with a legislative framework to govern micro health organizations, passed in 1997 [41, 42, 43]. In Mali, schemes are influenced by the presence of the Union Technique de la Mutualité Malienne (UTM), a technical agency that serves as an interface between the government and the mutual movement in Mali, that helps community groups establish CBHF schemes that are tailored to local needs [34]. UTM gives support to all mutual groups who request technical assistance [41, 42, 43]. In 1999, UTM developed the “Health Guarantee” plan, its own financing product available in Bamako and a few regional cities that offered four different types of benefit packages to the population: small risks, catastrophic risks, combination of small and catastrophic risks, and private financing. Health Guarantee is administered at the central level and has an extensive marketing strategy nationwide. An internal evaluation carried out in 2000 revealed that UTM succeeded in developing a mutualist movement in Mali, but it failed to attract a large number of members despite its high operating and administrative costs. Critics of UTM also pointed out that premium rates for the Health Guarantee were not affordable to Mali’s large informal sector [41, 43]. In Burkina Faso, CBHF schemes were initially led by healthcare providers, but are increasingly community-run [34]. In Benin, locally elected leaders have been actively involved in CBHF schemes [34]. Since then, there has been a general exponential growth in these types of organizations throughout Africa—in particular in West Africa. In Mali, Burkina Faso, and Benin, the estimated number of CBHF schemes was 24 in 1997, 71 in 2000, 128 in 2003, and 282 in 2006 [34].

In 1999, the Rwandan Ministry of Health instituted a CBHF pilot test in three districts. In order to collect policy development improvements, the Ministry collaborated with the partnership healthcare providers. The initiative was started in order to protect the communities most vulnerable and at risk. To date, CBHF schemes in Rwanda have grown into one of the “largest experiments in community-based risk-sharing mechanisms in sub-Saharan Africa for health-related problems” [44]. Similarly, the Bamako Initiative, widely used in Tanzania through community financing, was also intended to reach the poorest citizens. Its aim pointed towards the revitalization of the “public sector healthcare delivery system by strengthening district management teams and capturing some of the resources [spent] on health” [45]. This particular approach recognizes the importance of community involvement and health service decentralization. In both Tanzania and Uganda, user fees have been associated with a decline in hospital and clinic use. Only private and mission sectors are currently charging in Uganda, which reduces the prima facie case for social health insurance to protect against financial risk and catastrophic costs. In 1967, Nigeria passed its original legislation for the establishment of a government-operated health financing scheme—the system of National Hospital Financing Fund (NHIF), to cover hospital services. Under this system, NHIF operates as a traditional financing plan which serves as a third-party payer to certified institutions on a fee-for-service basis for inpatient drugs and medical services given to NHIF insured persons. While NHIF has successfully pooled risks for a significant portion of middle and upper-middle income Nigerians, it has not been able to expand its coverage to include more of the population, especially low-income earners.

### Challenges of CBHF in Africa

While the growth rate of these schemes has been impressive, it is rather unfortunate that it can only insure a small proportion of every administrative population. For example, the estimated number of beneficiaries in Mali, Burkina Faso, and Benin in 2006 was a little over half a million people out of a combined population of about 36 million in the three countries [34]. Although there does not appear to be a recent, systematic, cross-national inventory, available data indicates that there may be 1000 or more CBHF schemes in West Africa [10, 34]. Most of such schemes in sub-Saharan Africa have relatively few members, with 95% of the schemes having less than 1000 members [10]. While their small sizes limit the amount of risk-pooling, small CBHF schemes also have the benefit of having members that know each other and are actively involved in the management of the scheme [34].

Another main challenge of CBHF in Africa is the issue of equity. The Tanzanian bill that established national health financing for civil servants was mandatory and designed to cover employees, spouses, and children as legal dependents [46]. Preliminary assessment of the use of health financing among teachers has concluded that while the idea is sound, its implementation has been thwarted. For example, it has been documented that some healthcare providers are unaware of both this particular program and the existing payment models [47]. CBHF are more common in West Africa than in Central or East Africa. In some countries, these new schemes are mainly an urban phenomenon—such as in the Ivory Coast and Tanzania—whereas in other states, they are predominantly covering people in rural areas, e.g., Uganda, Ghana, and Benin. In Senegal, CBHF has a long tradition especially in the Thiès region in which more than 15 schemes in urban and rural areas are operating [48]. In the Democratic Republic of Congo, CBHF schemes mainly came to fruition in the second half of the 1980s [32]. The reason for this relatively early departure was the virtual stop of government funding for healthcare in the mid-1980s and the resulting need to rely on other sources of finance. Separate from equity, a similar challenge is the growing number of individuals who join the scheme only in the time of need, i.e., during illness to minimize medical expenditure without prior commitment or accountability. To ameliorate this, Huber et al. [49] suggest that interested members should be required to go through a waiting period to show they are genuinely interested before receiving benefits to avoid individuals joining the scheme at the time of illness to reduce their medical costs.

Having examined the benefits and the challenges of CBHF, it is important to take proactive steps to ensure sustainability. One way to do this, is to integrate CBHF within the larger health system of a given country [15]. Care must be taken to avoid such schemes from being viewed as a replacement for government-funded public healthcare [49]. As a result, the role of the government and its development partners must be strategic and coordinated [50]. The government should not establish and manage CBHF schemes, but should rather assume a stewardship role, enable an environment conducive to CBHF, and develop appropriate legislative frameworks [49]. At length, if the CBHF model sees an uptake, strategies to scale-up the model in a systematic fashion should be developed and drawn upon from the operationalization of existing schemes. CBHF schemes in Africa are, generally, initiated by private non-for-profit healthcare providers such as church-related organizations found in Nigeria, Ghana, Senegal, and the Democratic Republic of Congo. At the national level, several African countries have developed legal frameworks for CBHF implementation (e.g., Ghana and Senegal)—even making membership mandatory (e.g., Rwanda and Tanzania). Moreover, maturation of the CBHF movement has evolved at the bi-annual meeting held throughout the continent, i.e., via the use of managerial tools to improve uptake and means to structure the relationship of CBHF with other actors in the system—especially healthcare providers—and strategies to smoothen the integration of CBHF locally. Moreover, the CBHF movement not only has acquired important managerial applicability, it increasingly displays a systematic vision on healthcare delivery which African countries find extremely favorable.

### Socio-demographic factors affecting CBHF uptake

It is apparent that CBHF schemes are viewed by many as a promising new tool for health system improvement for rural populations in low-income countries, particularly in sub-Saharan Africa [35]. It is a means of providing financing coverage for rural communities unlikely to benefit immediately from either a social or private health financing system [51]. Hence, the schemes have the advantage of dissociating the time of payment from the time of service use, which is clearly better adapted than user fees to the seasonal fluctuations of revenue and expenditure flows of households. It is important to examine if CBHF and willingness to pay (WTP) for healthcare are influenced by socio-demographic factors such as age, gender, education, income, and other socio-cultural factors. A study by Dong et al. [52] on differential willingness of household heads to pay CBHF premiums, i.e., for themselves and other household members, found that age of household head was a negative coefficient and significantly influenced individual WTP and WTP per capita, while male gender and education had the expected positive associations. The study also showed that single marital status had a positive association and significantly influenced WTP per capita. The same study indicated that religion and disease episode did not have a statistically significant impact. As such, elements of household income and expenditure in a six-month block (i.e., on household members) both had a positive impact on WTP. Greater distance to the health facility had the expected negative association, reducing individual WTP and WTP per capita, although it was only statistically significant in the latter case [52]. These findings imply that the poor and the elderly are vulnerable groups that need to be taken into consideration when determining the implementation and design of CBHF schemes.

Furthermore, the prevailing concept of illness and risk make it very relevant to whether a household decides to purchase health financing or not. Cultural habits in dealing with the risk of illness can influence the demand for CBHF, e.g., in rural Benin, people commonly put money aside for unpredictable events like marriages and funerals but believe that saving money for eventual healthcare costs is equivalent to wishing oneself will become disease-stricken [53]. Fortunately, this attitude has changed following a CBHF scheme that had come into existence in the 1990s [53]. Solidarity (i.e., at the unity level) in a community is another strong factor for uptake of CBHF. That is, if solidarity is strong, people will not worry as much if benefits paid are accrued to themselves or other community members. For example, members of the Bwamanda scheme in the Democratic Republic of Congo expressed the opinion that if they did not need healthcare for themselves, at least they had done something good for the community by contributing to the financing fund [32, 34]. Creese and Bennett [35] further elucidate that the degree of solidarity and mutual trust is probably higher in homogeneous, close-knit communities than in scattered and diverse populations comprising of people from different ethnic origin, religion, and culture. This further can complicate the use of CBHF schemes and their design. Correspondingly, the use of the wealth index (i.e., as measured by income) is another key variable determining uptake of CBHF in Africa.

Widespread poverty among potential members can be a serious obstacle. If people are struggling daily to survive, they are less willing to pay financing premiums in advance in order to use services at a later point in time. A positive impact of health financing on equity and access must be questioned if a large proportion of the population cannot even afford CBHF membership. Social exclusion may persist even if barriers to health access is reduced for part of the population, and exemption mechanisms for the poorest—e.g., sliding scales for premiums—are enacted as a possible remedy [36]. After or before the introduction of health financing, rising income levels should be used to attract members and elucidate the potential benefits of CBHF. For instance, a flexible local economy has been cited as one of the factors contributing to the success of the Bwamanda scheme in the Democratic Republic of Congo that reached approximately 60% coverage of the target population and achieved approximately 80% recovery of hospital running costs [32, 35]. The examples of CBHF schemes throughout Africa, and especially in sub-Saharan Africa, depict socio-demographic factors as important performance and formulation indicators [23, 33, 42, 44]. To better evaluate and assist the health status of these countries, CBHF case research from the slums of Awka, Anambra State, Nigeria, is used to mitigate and show a gap in its applicability.

## Methods

A cross-sectional research design is adopted through a descriptive analysis to evaluate the presence and benefits of CBHF in Awka, Anambra State, Nigeria. The study adopted a quantitative method to gather needed descriptive data on the socio-demographic characteristics of the selected residents in the city of Awka who partake in CBHF schemes or otherwise. The data was collected from different parts of slums in the city which allowed for the generation of descriptive data. A descriptive survey generated statistical information on the socio-demographic features of participants of CBHF schemes among the residents of Awka. The study focused on the 12,246 JBT members with Jamii Bora Health Insurance (JBHI) located in the Awka branch [39]. The study applied the following formula, i.e., Eq. 1, to determine sample size selection [54, 55]. A sample size of *n* = 372 was calculated.

1 $$n=\frac{z^2\ast p\ast \left(1-p\right)/{e}^2}{1+\left({z}^2\ast p\ast \left(1-p\right)/\left({e}^2\ast N\right)\right)\ }$$

Where: *n* = sample size, *z* = z-score associated with a 95% level of confidence, *p* = sample proportion, *e* = margin of error, and *N* = population size (i.e., 12,246).

The study then adopted a stratified sampling method, which is commonly used to produce a precise estimate of characteristics of a whole population [56]. It is also used to ensure inclusion of the sampled items which otherwise can be omitted entirely by other sampling methods [56–58]. The method was used because the target population is divided into thirteen villages—labelled as strata (Table 1). For each stratum, the method of proportional allocation was used to keep the different stratum proportional to the size of the overall strata. Proportional allocation was considered the most efficient and an optimal approach since the population was assumed to be homogenous. There was no difference in the stratum variance since the population samples, measured for characteristic performance, came from a homogenous pool. The study randomly identified respondents in each stratum which ensured that all the villages and individuals in the villages (i.e., slums) would be included in the sampling procedure and, hence, stood an equal chance of inclusion.

**Table 1:** Sample frame

Strata number	Strata	Population^a^	Population (%)	Sample size	
				Insured	Non-insured
1	3A village	4059	5.05	10	10
2	3B village	7433	9.26	17	17
3	3C village	5316	6.62	12	12
4	4A village	18,776	23.38	44	44
5	4B village	5681	7.07	13	13
6	Gitathuru village	3737	4.65	8	8
7	Kiamutissya village	5825	7.25	14	14
8	Kosovo village	8085	10.00	18	18
9	Kwa Kariuki village	5290	6.59	13	13
10	Mabatini village	1160	1.44	3	3
11	Mashimoni village	4478	5.58	10	10
12	No. 10 village	2594	3.23	6	6
13	No. 2 village	7875	9.81	18	18
(*N*)	*Total population*	*80,309*	*100.00*	*186 +*	*186 = 372*

^a^sourced from the 2009 population census

A three-week pilot questionnaire was conducted with 30 sampled members to test for questionnaire issues (e.g., general reasoning, understanding, and format) and validity of the questions. Questionnaires were then officially administered to sampled members for the purpose of gathering study data. Questionnaires with guiding instructions, i.e., for the purposes of understanding the task, were distributed to the identified population by the authors and a number of research assistants. The respondents who received the questionnaires were made up of slum dwellers in the city of Awka who had previous knowledge about the CBHF scheme and whether they have an uptake or not in it.

To further strengthen the research, a pilot study was also undertaken in the slums of Kibera, Nairobi, Kenya, which has a similar JBHI scheme. This assisted the researchers by testing both the research instruments and the ability of the research assistants to administer the instruments. Moreover, members of the JBHI scheme in Kibera were sampled and interviewed which helped identify gaps in the instruments. A total of 50 respondents were included in the pilot study, validating the data collection method and the comparison of data with existing information in relevant offices and studies. This was done through cross-checking the information gathered with the already existing data in relevant institutions and through observation—ensuring data consistency and accurateness. In testing for reliability, piloting of the instrument was necessary. The instrument was tested using the split half method before the study was fully approved. In this case, the instrument was found to be resulting in inconsistencies which required necessary adjustments to ensure that they measured up to the required standards. This was done through simplifying some of the language in the questionnaires, reframing the questions, and restructuring the questionnaire. Guiding instructions were also rephrased, as necessary, to ensure that the instruments gave reliable information and what was desired in the study.

Descriptive statistics was applied to analyze the gathered data. After the data was collected and before the analysis was done; the data was first refined to eliminate any undesired or unwanted information that could have made the analysis problematic. This was done through organizing and editing the data to remove any repetitions, inconsistencies, errors, and anything not well understood as presented by the respondents. This was followed by coding the data to establish how possible answers would be treated by assigning them with numerical values. The final dataset was then stored in both soft and hard copies for reference during the analysis process. The data was tabulated and classified as per the characteristics observed and then analyzed in its own class using frequency tables. The data were analyzed using SPSS Statistics Version 27 and Microsoft Excel 2019 software packages. Similar studies of this nature in Africa [9, 10, 16], Asia [6, 12, 14], and Europe [11] have used this same method and have achieved sound results; as such, the reliability of the scientific method is validated and established.

Prior to the commencement of the study, verbal authorization was obtained from all the relevant authorities for conformity and to ensure that the study would not be discontinued. First, the authority from the University of Nairobi, Nairobi, Kenya, was given to commence the study as well as assistance from the University to seek the proper permissions within Kenya. Permission to commence the study was also sought from JBT which is the institution in charge of the CBHF scheme in the study area. The research further acquired authorization from the community health project’s Mathare Clinic which is the JBHI accredited hospital in Awka. Moreover, verbal consent from all respondents through talking and explaining to them the purpose of the study was received. Confidentiality was guaranteed and respondents are not identified in any way. The study was approved by the National Open University of Nigeria (NOUN) Ethical Committee and complied with the ethics of scientific research described in the NOUN Charter of Ethics and Ethical Principles in-line with the Declaration of Helsinki and other applicable ethical principles and legislation in Nigeria. Ethical approval was granted by the NOUN Research Administration and Advancement, Research Ethics Sector, Ethical Committee.

## Results

The study aims to examine the extent of changes in the socio-demographic characteristics of residents who are involved in the uptake of CBHF schemes in the city of Awka. A total of 372 questionnaires were distributed to selected respondents in the city which were all filled and returned with a 100% response rate. This was (clearly) an acceptable rate and was attributed to the fact that the questionnaires were administered mostly during the weekend and were physically dropped off to the respondents and collected on an agreed date. During the interaction with the respondents, fieldwork notetaking was performed in which observation and additional face-to-face dialogue was reported on. Additional questioning included the average number of times respondents were hospitalization annually and their general perspective (i.e., attitude) to the JBHI scheme, its service availability and reliability, and why they were happy (or not) with the scheme and how it could be improved. Table 2 illustrates the socio-demographic characteristics of the selected residents of Awka who participated in the study. The result shows more youth are non-insured (i.e., 18–35 years) compared with those in other age groups. For example, out of all the respondents between the ages of 18–35 years, 33.8% of them were insured while 46.7% were not. The respondents aged 36–50 years old indicated the largest percentage of the insured at 38.3%. The results in terms of gender distribution show there were more insured women in comparison to men. Among all the insured respondents, 38.1% of them were male while 61.9% were female. Whereas, among all non-insured respondents, 66.7% were male and 33.3% were female. This is significant since, in general, women are more interested and participate in community-based organizations in Nigeria than their male counterpart and, thus, get access to relevant information for better health-seeking behaviors [59–61].

**Table 2:** Socio-demographic characteristics of the respondents and health insurance uptake in Awka, Anambra State, Nigeria

Socio-demographic characteristics	Responses^a^					
	Insured		Non-insured		Total	
	%	*N*	%	*N*	%	*N*
Age groups						
18–35 years	33.8	63	46.7	87	40.3	75
36–50 years	40.0	74	38.3	71	39.2	73
Over 50 years	26.2	49	15.0	28	20.6	38
Gender						
Male	38.1	71	33.3	62	35.7	66
Female	61.9	115	66.7	124	64.3	120
Marital status						
Married	73.8	137	64.3	120	69.1	128
Unmarried	26.2	49	35.7	66	31.0	58
Number of children						
0–2	38.6	72	40.5	75	39.6	74
3–5	50.0	93	49.5	92	49.8	93
6+	11.4	21	10.0	19	10.7	20
Religion						
Protestant	62.8	117	53.8	100	58.3	108
Catholic	22.4	42	32.9	61	27.7	51
Islam	14.8	28	13.3	25	14.1	26
Income level						
Lowest	32.4	60	46.7	87	39.6	74
Low	34.3	64	30.0	56	32.1	60
Modest	20.0	37	18.1	34	19.0	35
High	13.3	25	5.2	10	9.3	17
Education level						
No formal education	9.5	18	5.2	10	7.4	14
Primary school	33.8	63	39.7	74	36.7	68
Secondary school and higher	56.7	105	55.1	102	56.0	104

^a^*N* = absolute number

In addition, the study examines the relationship between specific socio-demographic factors and the level of resident participation within the CBHF schemes. For example, how marital status relates to the level of insurance status of the respondents. The results reveal the proportion of those insured was higher among the married (i.e., 73.8%) than the unmarried (i.e., 26.2%). This confirmed the assertions of previous studies which suggested that marital status had a statistically significant positive effect on health insurance ownership [35, 36]. Married persons are more likely to have insurance coverage than those who are single, separated, or divorced. The study also examined married couples with children vis-à-vis the uptake of the insurance. The results show one of the reasons for joining the JBHI scheme among the insured respondents differed in-line with the number of children they had. The majority, i.e., 50.0%, of the insured respondents had three to five children while 38.6%, who had less than two children, joined JBHI scheme because of the hard economy they were experiencing. In comparison, 11.4% of the respondents who had more than six children said they joined the scheme so they could avoid out-of-pocket payments whenever family members are admitted into the hospital.

The study examines also the scope of social health insurance within the informal sector. Thus, it found that within the city of Awka’s slums, informal savings groups were common, and slum dwellers made monthly contributions—communally. The slum dwellers shared their contributions on a revolving basis as a member-based group to help cater for various needs, including funeral and healthcare expenses. This is necessary since urban slum dwellers in Nigeria are often being confronted with inadequate waste disposal methods that festers disease, which can easily turn into a local outbreak [62]. This result reflected in our data analysis since 66.7% of the insured respondents were from the lowest and low-income levels. On the other end of the spectrum, 33.3% of the insured respondents were from moderate- and high-income levels.

Similarly, the study sought to examine the religion of the respondents and their insurance status. The results show 62.8% of the insured respondents were Protestant and 22.4% were Catholic, while 14.8% of the respondents were from the Islamic faith. Likewise, 53.8% of the non-insured respondents were Protestant and the percentages for Catholic and Muslim were 32.9 and 13.3%, respectively. Participation in the JBHI scheme was significantly higher among respondents who have secondary school and higher education level (i.e., 56.7%). Those who had primary school education and are insured constituted 33.8%. There was a significantly low enrolment of the insurance among the respondents who had no formal education (i.e., 9.5%). The research underscored those with secondary school education level and above as more likely to use JBHI benefits than those with less education. With the exception of a few results, particularly the place of residence, most other results obtained in from this study validate or corroborate with findings from other similar studies [6, 9–12, 14, 16]. It can be stated, the drive and concern to have the option of a CBHF scheme has been found to be beneficial to low-income earners and tend to take it more serious than affluent households.

## Discussion

The data disclosed that more youth are non-insured compared to those in other age groups. It can also be observed that more women were insured. This could be attributed to the fact that more women are involved in organizing and being a part of community-based organizations throughout Nigeria [59–61] wherein there is easy access to information gathering and sharing. This concurred with the study by the Department of Gender and Social Development in which Olonade et al. [63] found that more women form and join organizations nationwide. Furthermore, joining JBHI was higher among those with secondary education level and higher. This finding is in tandem with findings from Ekman [64] who found that education levels are related with take up of health insurance programs in the developing world. Unfortunately, many low-income individuals are unfamiliar with the concept of insurance programs. The findings, however, disagree with Merlis [65] who found that awareness by the government is more correlated to uptake of health insurance policies compared to levels of education and income of urban slums dwellers. Most of the urban poor use either the public and faith-based hospitals for inpatient care, because, according to previous studies the main reasons for this choice, was the type of services offered, the perceived quality of care, and overall accessibility [66]. Moreover, the study found evidence that the number of children that a respondent had increased the likelihood of enrolling into the JBHI scheme. Respondents with six or more children were more likely to join JBHI to avoid out-of-pocket payments associated with medical expenses. This result showed that enrollment in the JBHI scheme was associated with perceived financial risk of accessing healthcare and number of children that a member had. The result revealed that the proportion of the insured was higher among the married (i.e., 73.8%) than the unmarried (i.e., 26.2%). These findings are consistent with the result obtained by others studies [67–69]. Harmon and Nolan [68] found that married couples may have a higher demand for health insurance due to the need to protect their children, higher combined income, and being more averse to the risk of catastrophic health expenditures than those who are single, separated, or divorced. A possible explanation for this finding is that when individuals get divorced, widowed, or separated, they may become financially vulnerable hence impacting their ability to make payments to the JBHI scheme.

The data disclosed that individuals in the lowest and low-income group are more likely to be enrolled in the JBHI scheme in comparison to others. This is supported by previous studies, suggesting that inclusion of the poorest was dependent on the design and implementation features of the scheme [70–78]. JBHI collects weekly premiums throughout the year, which makes payment of premiums affordable even for the very poorest households. The findings revealed that respondent perception of the quality of care provided by the contracted healthcare providers also influenced the decision to enroll—another similar finding from previous studies [15, 72, 74, 76–78]. In light of this, health resources and the ability to meet daily needs is worsening in Nigeria [33, 79]. Unfortunately, poverty is usually closely connected with poor health outcomes, “with the poorest of the poor having the worst health outcomes” [76], especially those in vulnerable situations such as the displaced persons in camps [80, 81]. In high-income countries, health insurance schemes (e.g., CBHF) are the norm, however, in developing countries such as in Nigeria, they should be considered still a work in progress as access to basic healthcare is not always easily achieved—especially for people from informal settlements. This paper highlighted case research that exemplified this shortfall in hope that situational reform can be made. It should be stressed, even though the study was based in Awka, the study findings are most likely reciprocal to other similarly situated areas in Nigeria and sub-Saharan Africa. To improve research in this field of work, further investigation is needed to examine additional measures of how CBHF factors influence individual CBHF uptake, how they impact on the treatment of non-communicable diseases, and what measures are needed to mitigate the complete utilization of CBHF-based schemes.

## Conclusion

The study demonstrates the key socio-demographic characteristics of the people who have uptake of CBHF in Awka, Anambra State, Nigeria. It was found that uptake of JBHI benefits was higher than in most other CBHF studies [67–71, 73]. It was also revealed that this could be attributed to the fact that JBHI has no per capita financial capping and does not require any co-payments to access healthcare. This is relevant since the study only focused on the JBT scheme in Nigeria—leaving out eight primary organizations that also operate in the country. Similar to the KCBHFA system, i.e., from Kenya, Nigeria’s CBHF schemes are sparsely divided throughout the country with varying levels of care. Overall, JBHI favored the members in the lower income quintiles who were more likely to use healthcare services covered by the JBT scheme. Insured members reported higher use of hospitalization care than the non-insured. This confirmed that prepayment schemes and the pooling of risk could reduce financial barriers to healthcare among the urban poor.

On the basis of these findings, the following three recommendations are suggested. First, since the majority of youth respondents have the lowest enrollment in CBHF, it would be recommended that a review of the insurance package made by CBHF be more attractive and suitable to younger people. This is a good idea, since it creates a strong generational commitment to sound healthcare and cyclic financial support of the system as a whole. Second, since 74% of the respondents said that service delivery at JBT could be improved by simply increasing the amount of hospitals covered under the scheme, it would be recommended that the national JBHI network expand their 90 approved hospitals so that their services can be more accessible to the community. This would, potentially, enlarge their cliental and, indirectly, improve hospitals and clinics requiring the JBHI standard. Third, the need for community sanitization should be prioritized in order to initiate a change of cultural beliefs and any misconception that inhibits enrollment of cliental into CBHF-based schemes. Disease and illness should be seen as somewhat of a random event that can hit anyone and, hence, preparedness via insurance should be highlighted at all levels of government. This study, through a detailed literature review, managed to piece together key CBHF historical components specific to sub-Saharan countries. It is therefore expected that it will aid, especially the bottom end of the economic ladder and the safety net necessary to meet healthcare requirements. Future study into socio-demographic factors is needed and should focalize on developing further support mechanisms such as employer-based health insurance, family health risk, and educative social and community planning.

## Data Availability

The data is available from the corresponding author on reasonable request.
